# Supplementary material for: Wearable smart sensor systems integrated on soft contact lenses for wireless ocular diagnostics
Source: Nat Commun. 2017 Apr 27;8:14997. doi: 10.1038/ncomms14997 (PMC5414034; doi:10.1038/ncomms14997)
Supplement: Supplementary Information — Supplementary Figures, Supplementary Table and Supplementary Note. [file ncomms14997-s1.pdf]

Supplementary Figures

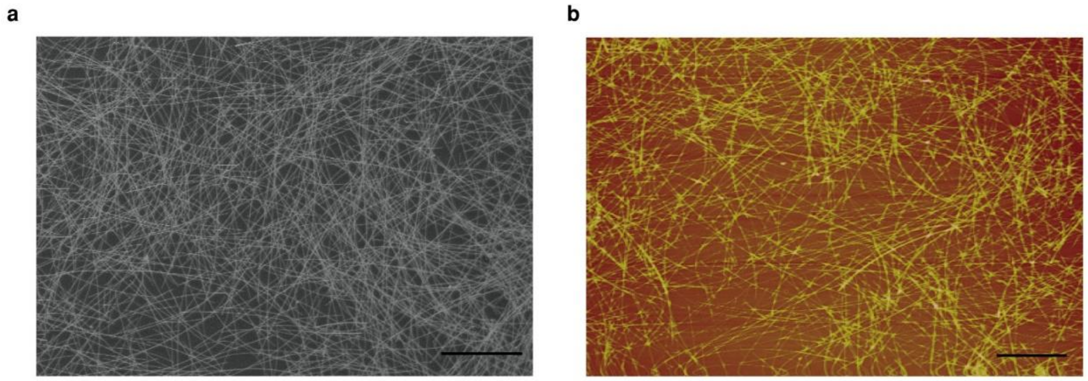

Supplementary Figure 1 | The diameter and length of AgNWs. (a) SEM image and **(b)** AFM image of AgNWs coated on a SiO_2/Si wafer at 500 rpm for 30 sec. The diameter and length of the AgNWs are averagely 30 (± 5) nm and 25 (± 5) µm, respectively. Scale bar, 10 µm.

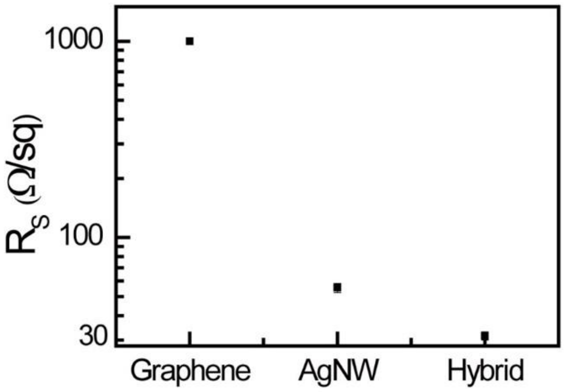

Supplementary Figure 2 | Sheet resistance of bare graphene, AgNW, and Hybrid. This hybrid structures had much lower sheet resistance (Rs) of about 30 Ω/sq than graphene synthesized by CVD (> 1 kΩ/sq) or AgNW.

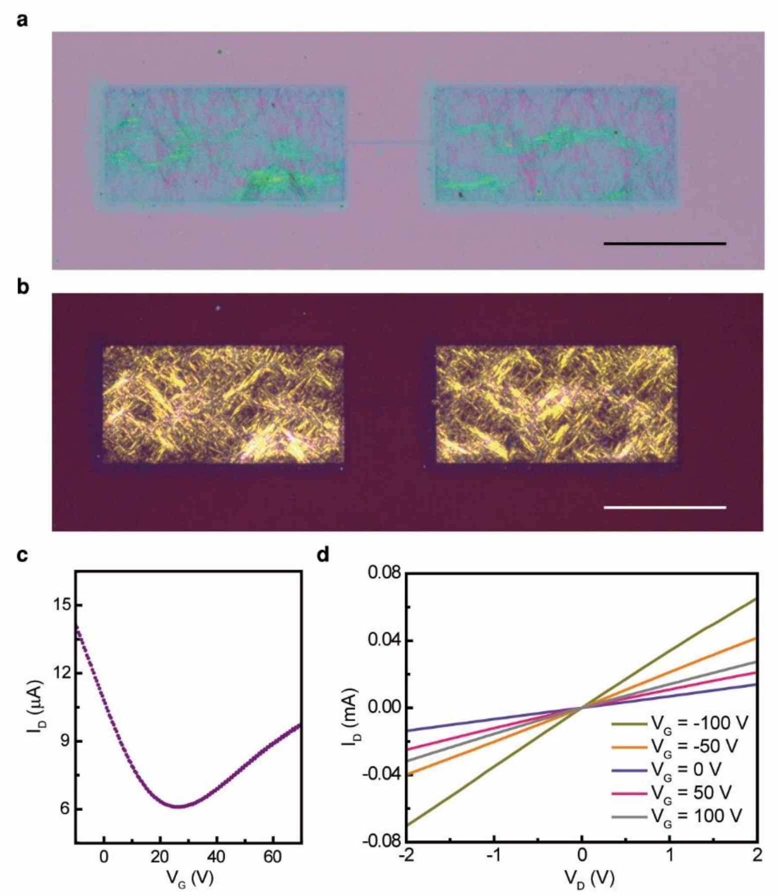

Supplementary Figure 3 | Optical micrograph and electrical properties of graphene FET. **(a)** Optical microscope image of graphene FET with graphene-AgNW hybrid electrodes in bright-field version and **(b)** dark-field version. The channel length and width of the device are 70 μm and 5 μm, respectively. Both black and white scale bars, 100 μm. **(c)** Transfer characteristics (I_D-V_G) of graphene FETs on SiO_2/Si substrate at V_D = 0.1 V. The channel length and width of the device: 70 μm and 5 μm, respectively. **(d)** Output characteristics (I_D-V_D) of the transistor at different gate voltages from -100 V to 100 V.

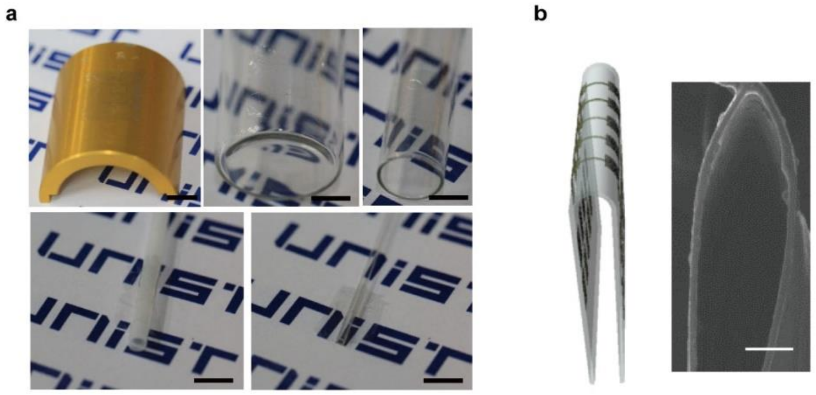

Supplementary Figure 4 | Bending and folding image of graphene-AgNW hybrid devices. **(a)** Photos of devices wrapped on various cylindrical supports with different radius. (**b)** Schematic illustration and SEM image revealing ultimate flexibility of hybrid devices that was folded in half. (Radius of curvature: ~3.1 μm). Scale bar, 10 μm.

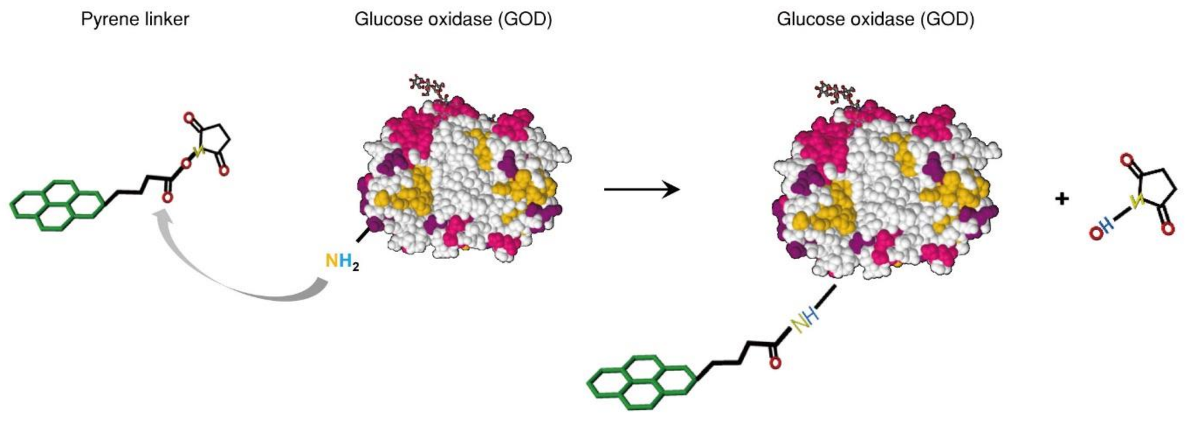

Supplementary Figure 5 | Amide bond between pyrene linker and glucose oxidase (GOD). The formation of amide bond was resulted with the nucleophilic substitution of N-hydroxysuccinimide by amine group on protein.

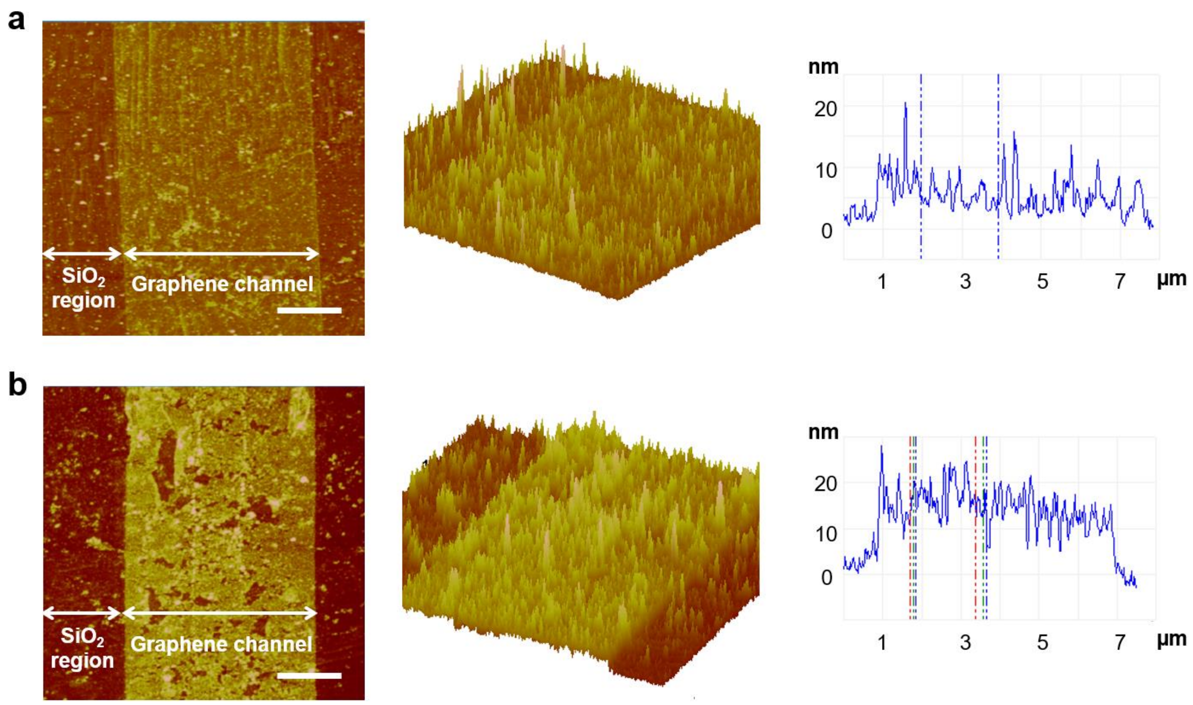

Supplementary Figure 6 | The AFM images before and after GOD coating. The AFM topologies of graphene channel **(a)** before and **(b)** after GOD coating. Scale bars, 2 μm.

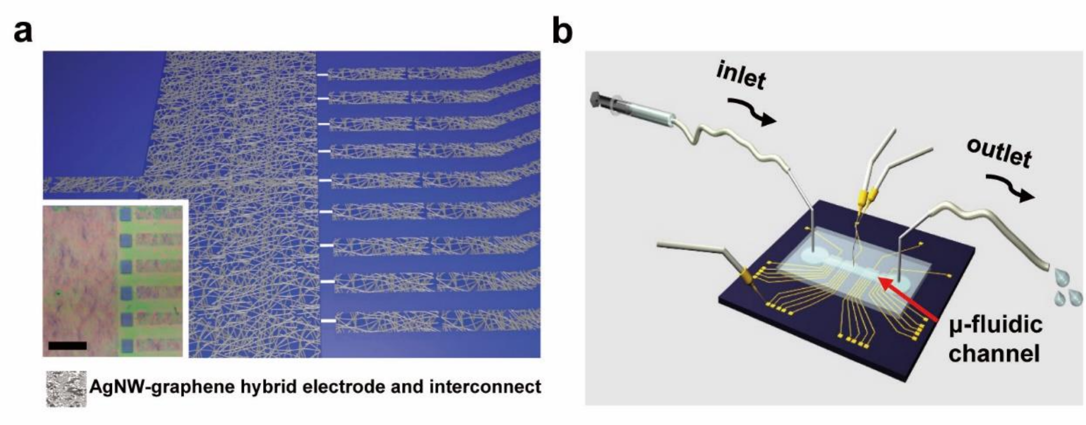

Supplementary Figure 7 | Schematic and optical images of a sensor. (a) This structure consists of block array of 9 graphene-FET sensors with the graphene-AgNW hybrid S/D electrodes and interconnects. Scale bar, 100 μm. **(b)** Illustration of the real-time glucose sensing using a PDMS based micro-fluidic channel.

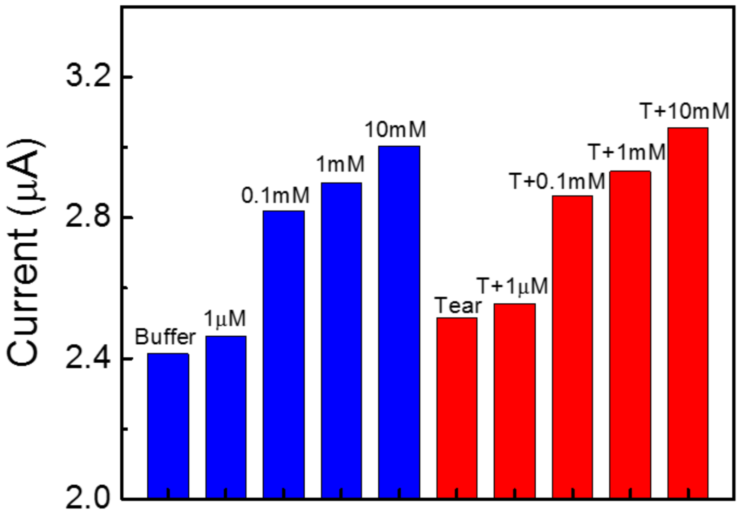

Supplementary Figure 8 | Detection of glucose in different solution environments. The current characteristics of the sensor in response to the glucose dissolved in buffer (blue) and artificial tears (red).

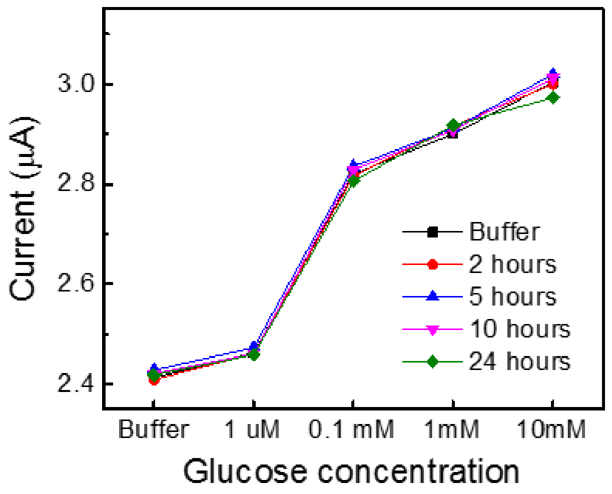

Supplementary Figure 9 | Stability of the glucose sensor. Calibration currents for various glucose concentration with the passage of time.

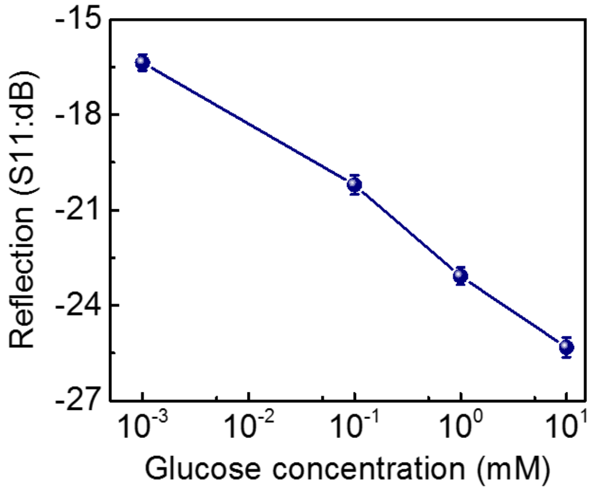

Supplementary Figure 10 | Calibration curve of wireless glucose sensor. The calibration curve generated by reflection coefficient and the glucose concentration from 1 μM to 10 mM.

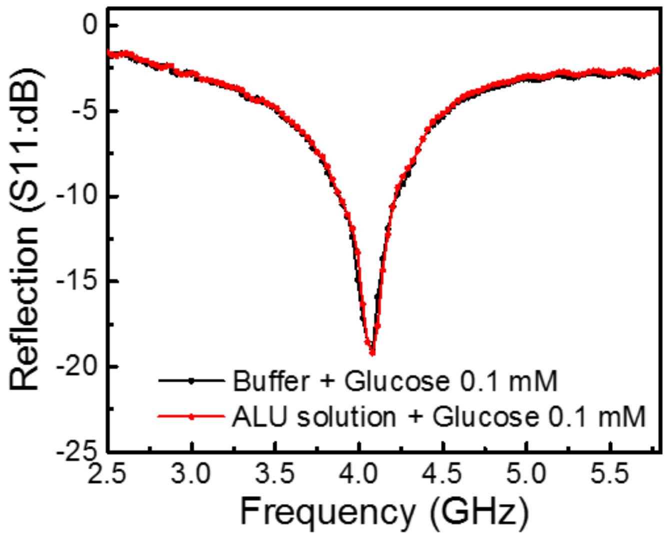

Supplementary Figure 11 | Selectivity of the glucose sensor. The reflection coefficient of the antenna at the resonance frequency in response to the glucose (0.1 mM) dissolved in buffer (black) and ALU solution (A: 50 μM ascorbic acid, L: 10 mM lactate, U: 10 mM urea) (red).

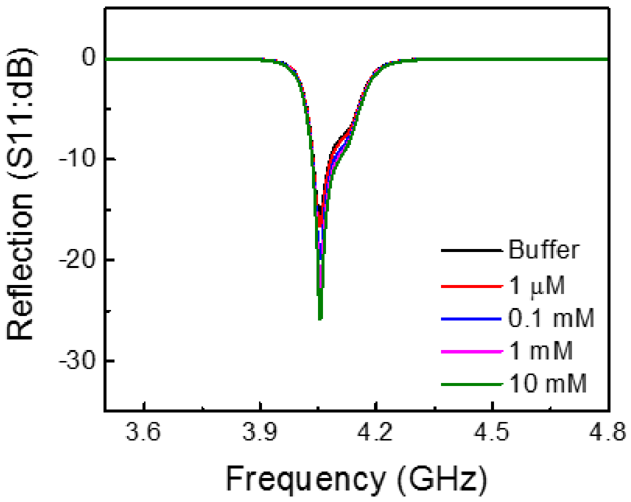

Supplementary Figure 12 | The simulation result of wireless glucose sensor. The simulated reflection value of glucose concentration from 1 μM to 10 mM.

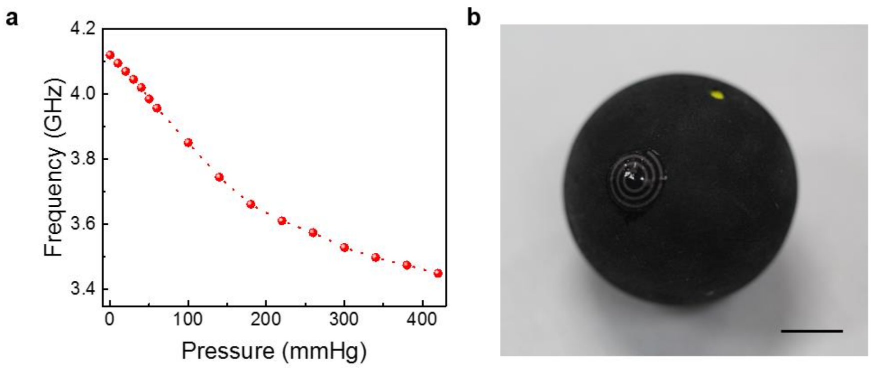

Supplementary Figure 13 | Frequency response of the pressure sensor using a ball. (a) The resonance frequency change in the wide range of pressure. **(b)** Photograph of the sensor on a ball. Scale bar, 1 cm. In this experiment, a ball was used instead of a bovine eye because this bovine eye is burst in the relatively big pressure regime.

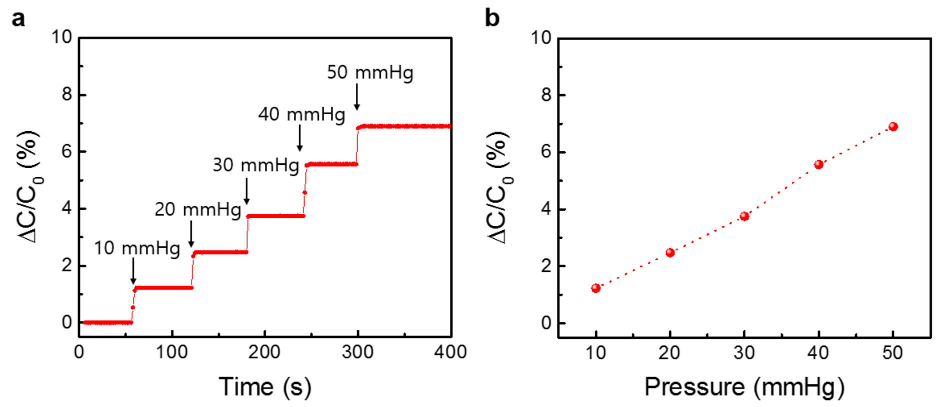

Supplementary Figure 14 | Capacitance response of the intraocular pressure sensor. (a) Normalized capacitance change of the intraocular pressure sensor in the physiological intraocular pressure range (0 - 50 mmHg). **(b)** Relative capacitance change by the applied intraocular pressure (0 - 50 mmHg).

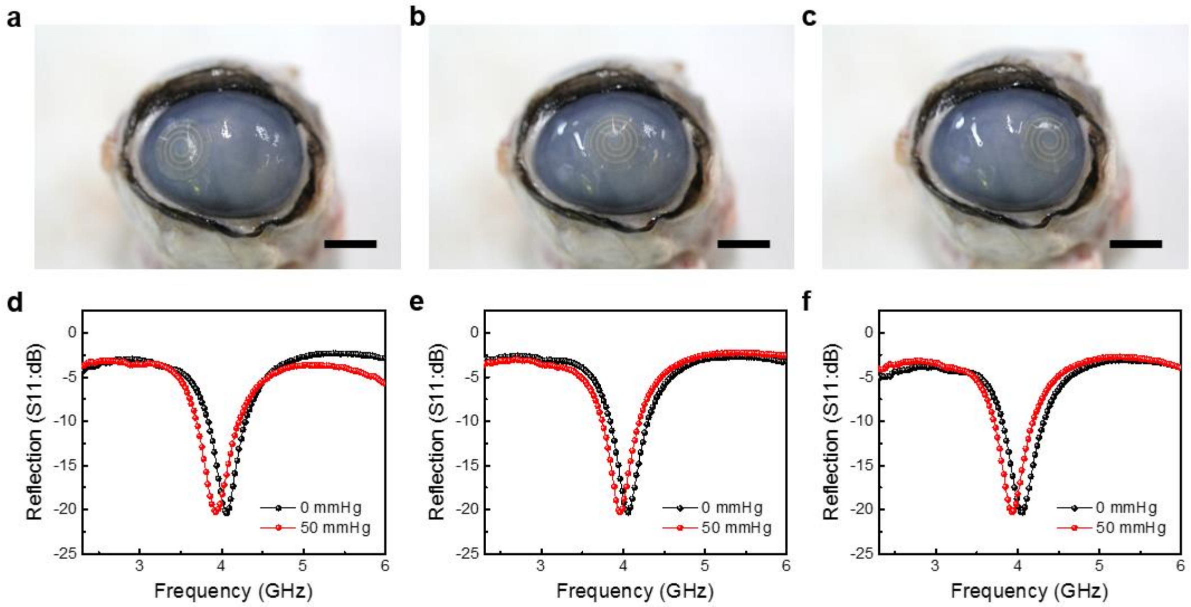

Supplementary Figure 15 | The effect of slippage on the frequency response of the intraocular pressure sensor. Photographs of the intraocular pressure sensor on the (a) left, (b) centre, and (c) right of the bovine eyeball. Scale bars, 1 cm. The frequency response of the sensor on the (d) left, (e) centre, and (f) right of the bovine eyeball at 0 mmHg and 50 mmHg.

Supplementary table

Ref.	Sensor	Novelty	Disadvantages
14	Strain gauge sensor	1) Fabrication of light and biocompatible Oxide based TFT sensor	1) Plastic lens-shaped substrate<br>2) Using the opaque metal (Ti/Au)<br>3) Single sensor<br>4) Physical connection between sensor and measurement equipment
15	Glucose sensor	1) Fabrication of ln_2O_3-based FET sensor using simple solution-processing procedure<br>2) Glucose or pH sensor	1) Transferred on the artificial eye<br>2) Using the opaque metal (Cr/Au)<br>3) Physical connection between sensor and measurement equipment
17	Intraocular pressure sensor	1) Fabrication of wireless intraocular pressure sensor	1) Silicone lens-shaped substrate<br>2) Using the opaque metal (Cu)<br>3) Single sensor
18	Glucose sensor	1) Fabrication of sensitive glucose sensor by immobilizing glucose oxidase	1) Lens-shaped PET substrate<br>2) Using the opaque metal (Cu)<br>3) Single sensor
Our results	Glucose and intraocular pressure sensor	1) Use of commercialized soft contact lenses<br>2) Fabrication of wireless sensor<br>3) Fabrication of flexible/stretchable and transparent sensor based on the graphene/AgNW structures<br>4) Multiplexed enabling simultaneous detection of both glucose and intraocular pressure	

Supplementary Table 1 | Comparison between this work and the previous reports.

Supplementary Note

Wireless sensing measurement

The reflection at the resonance frequency is inverse proportion to the electrical resistance in the graphene channel as resistive element of the circuit. The expression for which is, using the Kirchhoff's circuit laws,

$$\begin{bmatrix} I_1 \\ I_2 \\ I_3 \end{bmatrix} = \begin{bmatrix} Z_1 & j\omega M_{12} & 0 \\ j\omega M_{12} & Z_2 & j\omega M_{23} \\ 0 & j\omega M_{23} & Z_3 \end{bmatrix}^{-1} \begin{bmatrix} V_s \\ 0 \\ 0 \end{bmatrix} \quad (1)$$

$$Z_1 = R_{source} + R_r + j\left(\omega L_r - \frac{1}{\omega C_r}\right) \approx R_{source} \quad (2)$$

$$Z_2 = R_{antenna} + j\left(\omega L_{antenna} - \frac{1}{\omega C_{antenna}}\right) = R_{antenna} \quad (3)$$

$$Z_3 = R_{sensor} + j\left(\omega L_{sensor} - \frac{1}{\omega C_{sensor}}\right) = R_{sensor} \quad (4)$$

where L_x, C_x, M_{xy}, and Z_x (R_x)are the inductance, capacitance, coupling coefficient, and resistance of reader coil ($_s$), resonate receiver coil ($_2$), and load coil ($_3$), respectively.

$$V_s = Z_1 I_1 + j\omega M_{12} I_2 \quad (5)$$

$$0 = j\omega M_{12} I_1 + Z_2 I_2 + j\omega M_{23} I_3 \quad (6)$$

$$0 = j\omega M_{23} I_2 + Z_3 I_3 \rightarrow I_3 = -\frac{j\omega M_{23} I_2}{Z_3} \quad (7)$$

$$0 = \mathrm{j}\omega M_{12} I_1 + Z_2 I_2 + j\omega M_{23}\left(-\frac{j\omega M_{23} I_2}{Z_3}\right)$$

$$= \mathrm{j}\omega M_{12} I_1 + Z_2 I_2 + \frac{\omega^2 M_{23}^2 I_2}{Z_3}$$

$$= \mathrm{j}\omega M_{12} I_1 + \left(Z_2 + \frac{\omega^2 M_{23}^2}{Z_3}\right) I_2 \quad (8)$$

$$\rightarrow I_2 = \left(\frac{-j\omega M_{12}}{Z_2 + \frac{\omega^2 M_{23}^2}{Z_3}}\right) I_1 \quad (9)$$

$$V_s = Z_1 I_1 + j\omega M_{12} I_2 = Z_1 I_1 + j\omega M_{12} \left(\frac{-j\omega M_{12}}{Z_2 + \frac{\omega^2 M_{23}^2}{Z_3}} \right) I_1$$

$$= Z_1 I_1 + \left(\frac{\omega^2 M_{12}^2}{Z_2 + \frac{\omega^2 M_{23}^2}{Z_3}} \right) I_1 \qquad (10)$$

$$Z_{in} = \frac{V_s}{I_1} = Z_1 + \frac{\omega^2 M_{12}^2}{Z_2 + \frac{\omega^2 M_{23}^2}{Z_3}} = \frac{Z_1 Z_2 Z_3 + \omega^2 M_{23}^2 Z_1 + \omega^2 M_{12}^2 Z_3}{Z_2 Z_3 + \omega^2 M_{23}^2} \qquad (11)$$

Substituting this variable in the following expression, the S11 is proportional to the channel resistance (Z_3) by equation (12),

$$S_{11} = \frac{Z_{in} - R_{source}}{Z_{in} + R_{source}} = \frac{\omega^2 M_{12}^2}{2 Z_1 Z_2 + \frac{2\omega^2 M_{23}^2 Z_1}{Z_3} + \omega^2 M_{12}^2} \qquad (12)$$
